# Mitochondrial and Nuclear Markers Reveal Contrasting Patterns of Genetic Diversity in the Red Palm Weevil (*Rhynchophorus ferrugineus*) from Qassim Province, Saudi Arabia

**DOI:** 10.3390/life16071200

**Published:** 2026-07-20

**Authors:** Saleh S. Alhewairini, Medhat Rehan, Mohamed I. Motawei, Mahmoud Alazzazy, Nagdy F. Abdel-Baky

**Affiliations:** Department of Plant Protection, College of Agriculture and Food, Qassim University, Buraydah 51452, Saudi Arabia

**Keywords:** red palm weevil, genetic diversity, COI, ITS, haplotypes, molecular markers, phylogenetic analysis, Integrated Pest Management (IPM), sustainability

## Abstract

The red palm weevil, *Rhynchophorus ferrugineus (Olivier)*, is among the most destructive invasive pests of date palms worldwide. In this study, mitochondrial cytochrome c oxidase subunit I (COI) and nuclear internal transcribed spacer (ITS) markers were analyzed in parallel to comparatively assess genetic diversity and haplotype variation in *R. ferrugineus* populations from Qassim Province, Saudi Arabia. Sequencing success rates reached 96.5% and 93.0% for COI and ITS, respectively. COI sequences exhibited very low nucleotide divergence among Qassim specimens (0.0–0.0077), indicating a highly conserved mitochondrial background and close phylogenetic similarity (*p*-distance = 0.0–0.0078) with an Egyptian reference haplotype (GU581319) and the reference *R. ferrugineus* mitochondrion (KT428893). In contrast, ITS analyses revealed substantially greater nuclear variation, identifying multiple haplotype groups with divergence levels of 10–19%. Haplotype diversity was higher in ITS (Hd = 0.876 ± 0.041) than in COI (Hd = 0.663 ± 0.068), while nucleotide diversity in ITS (π = 0.0387 ± 0.0013) was 36.9-fold greater than in COI (π = 0.00105 ± 0.00025; Z = 27.66, *p* < 0.001). Phylogenetic reconstruction showed greater population structuring in ITS than in COI. Integrated mitochondrial and nuclear markers improve genetic resolution and species identification in *R. ferrugineus*.

## 1. Introduction

*Rhynchophorus ferrugineus* (Olivier), the red palm weevil (RPW) (Coleoptera; Family: Dryophthoridae, formerly Curculionidae), is one of the most destructive pests of date and coconut palms worldwide, causing severe internal damage and substantial economic losses. Since its original distribution in South and Southeast Asia, *R. ferrugineus* has expanded across the Middle East, North Africa, the Mediterranean Basin, and parts of Europe, largely facilitated by the international movement of infested palm offshoots and planting material [1,2,3,4,5]. In arid and semi-arid regions such as Saudi Arabia, date palms are of major ecological, economic, and cultural importance, making RPW infestations a significant agricultural concern [4,5,6]. The concealed feeding behavior of larvae within palm trunks allows infestations to progress undetected until advanced stages, often leading to structural failure and palm mortality [6,7].

Despite long-standing management efforts, effective control of *R. ferrugineus* remains challenging [3]. Chemical insecticides, particularly systemic formulations applied via trunk injection or soil drenching, are widely used control measures in infested regions [8,9]. However, their effectiveness is often inconsistent due to limitations in application techniques, reduced penetration into internal palm tissues, and variability in palm physiology and environmental conditions [10,11,12,13]. These factors, together with differences in orchard management practices, can influence treatment outcomes and complicate control strategies [12,13].

Morphological variation among adult *R. ferrugineus* individuals, particularly in coloration and pronotal patterns, has been widely reported across its distribution range and may overlap with traits observed in closely related species such as *R. vulneratus* and *R. bilineatus* [2,14,15]. However, morphological variability alone is insufficient for reliable taxonomic discrimination, and integrative approaches combining morphological and molecular data are required for accurate species identification and population monitoring [16,17,18]. This is particularly important in regions where multiple *Rhynchophorus* taxa may show overlapping external characteristics.

Molecular markers have become essential tools for assessing genetic diversity and evolutionary relationships in invasive insect species. Mitochondrial markers such as cytochrome oxidase subunit I (COI) are widely used for species identification and broad-scale genetic screening, although they may show limited variability in some invasive populations [2,19,20]. Nuclear markers, including the internal transcribed spacer (ITS) region, provide complementary information and may reveal additional levels of sequence variation not detected by mitochondrial loci. The nuclear ITS region was incorporated as a complementary marker to the mitochondrial COI gene to provide a broader and more robust assessment of genetic diversity within *R. ferrugineus* populations [21,22,23,24,25]. Mitochondrial and nuclear markers frequently capture different dimensions of evolutionary and population-level variation due to differences in inheritance patterns, mutation rates, and genomic organization. In the present study, the comparative use of ITS proved particularly informative, revealing substantially greater genetic variability, broader haplotype differentiation, and stronger population structuring than that detected by COI alone [21,22,23,24,25]. These findings highlight the potential of nuclear markers to uncover hidden patterns of genetic variation that may remain undetected when relying exclusively on mitochondrial datasets. ITS has been extensively applied in insect molecular studies because of its relatively high sequence variability and effectiveness in resolving intra- and inter-population differences among closely related lineages [21,24,25,26]. However, ITS also possesses recognized limitations, particularly in Coleoptera, because it represents a multicopy nuclear region that may be affected by incomplete concerted evolution, intra-genomic sequence heterogeneity, and possible amplification of paralogous or pseudogene-associated copies. Therefore, interpretations based on ITS variation should be considered cautiously and ideally integrated with complementary molecular evidence to achieve more reliable inference of genetic diversity and evolutionary relationships [26,27,28]. Therefore, the combined mitochondrial–nuclear approaches are increasingly recommended for more comprehensive assessments of genetic diversity [2,21,22,23,24,25,26].

Understanding genetic variation is important for interpreting population history and variability in invasive insects. However, inferences about population connectivity or gene exchange require careful interpretation and robust analytical frameworks. In *R. ferrugineus*, dispersal is influenced both by natural movement and, more significantly, by human-mediated transport of infested planting material, which can shape spatial genetic patterns in a complex manner [27,28,29,30].

In Saudi Arabia, *R. ferrugineus* has been established for several decades [2,3,5], yet detailed comparative studies of mitochondrial and nuclear genetic variation at regional scales remain limited. In particular, the extent to which different molecular markers capture variation within local populations has not been fully evaluated [26,27,28,29,30].

Despite the extensive use of mitochondrial markers in population genetic studies of the red palm weevil, their capacity to accurately represent genome-wide genetic variation remains inherently constrained due to maternal inheritance and reduced effective population size. In contrast, biparentally inherited nuclear markers, particularly the internal transcribed spacer 2 (ITS2), provide a more comprehensive and evolutionarily informative assessment of genomic diversity and population structure. Given these contrasting biological properties of mitochondrial and nuclear genomes, direct comparisons between marker systems are essential for evaluating the extent to which mitochondrial datasets accurately capture underlying genetic diversity. We therefore hypothesized that mitochondrial cytochrome oxidase I COI-based analyses substantially underestimate the true magnitude of genetic diversity compared with nuclear-derived estimates. To evaluate this hypothesis, we performed a quantitative comparative analysis of diversity indices generated from both mitochondrial and nuclear datasets across multiple populations. This integrative framework was designed to identify potential discrepancies between marker systems and to enhance the resolution, accuracy, and interpretative power of population genetic inferences in *R. ferrugineus*.

## 2. Materials and Methods

### 2.1. Collection of Rhynchophorus ferrugineus Specimens

**Figure 1:**
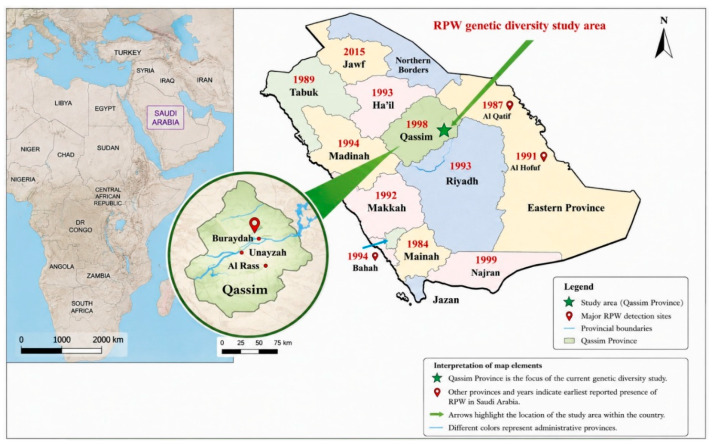
Geographic location of Qassim Province, Saudi Arabia, and the timeline of *R. ferrugineus* introduction and spread in the country (adapted from Abdel-Banat et al. [5].

Qassim Province (26°12′28.174″ N, 43°29′1.457″ E) is located in central Saudi Arabia (SA), approximately 400 km northwest of Riyadh. The province is bordered by Riyadh Province to the south and east, Ha’il Province to the north, and Al-Madinah Province to the west (Figure 1). Qassim is one of the most important agricultural regions in Saudi Arabia, characterized by extensive date palm cultivation, high cultivar diversity, and intensive agricultural activity that supports regional food production and economic stability [31,32].

**Figure 2:**
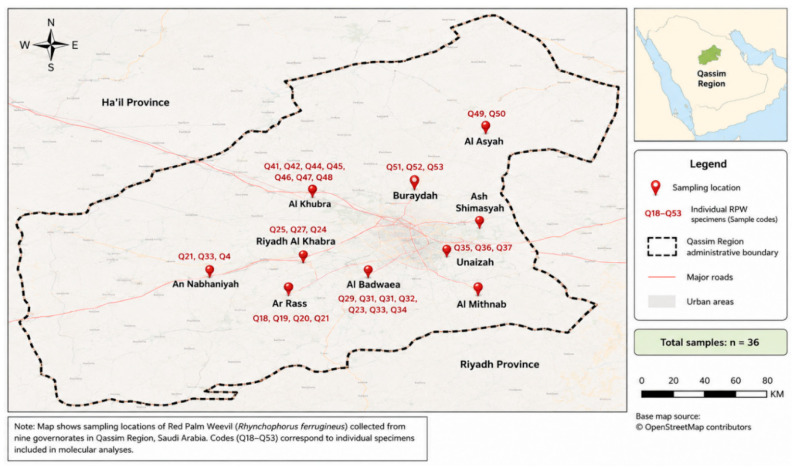
Sampling locations of *R. ferrugineus* used for genetic diversity analysis in Qassim Province, Saudi Arabia.

Nine major date palm cultivation sites distributed across Qassim were selected to capture a broad spatial representation of *R. ferrugineus* occurrence (Figure 2). Sampling was conducted during 2021, targeting adult weevils collected directly from infested palm trees. Initial identification of specimens was performed using established morphological diagnostic characters, particularly pronotal spot patterns and external coloration features, following standard taxonomic keys for the genus *Rhynchophorus* [2,15,33].

**Figure 3:**
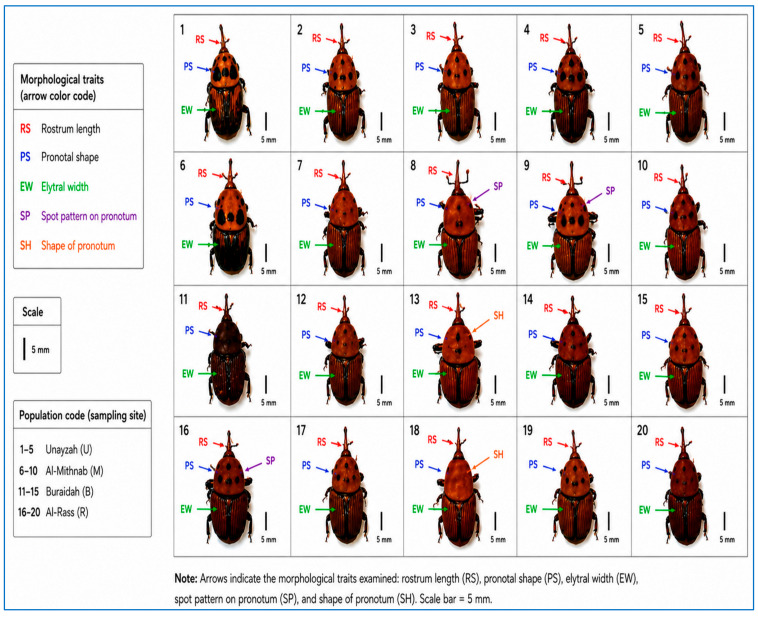
Morphological variation in *R. ferrugineus* populations in Qassim Province, Saudi Arabia.

A total of 250 adults *R. ferrugineus* individuals were collected from nine geographically separated date palm cultivation sites across Qassim Province, Saudi Arabia. All collected specimens were subjected to systematic morphological examination immediately after collection (Figure 3). The term “morphological groups” refers to descriptive phenotypic categories (Morphotypes) established according to observable external characters, including body coloration, pronotal spot patterns, and related morphological traits. These categories were used solely for classification and organizational purposes to represent the range of observed phenotypic variation and do not imply genetically distinct lineages, biological forms, or population subdivisions [2,34]. The morphological survey of the full collection revealed substantial phenotypic variability, which was categorized into 57 distinct morphological groups. It is important to clarify that these 57 categories represent phenotypic classification units derived from the complete field collection (*n* ≈ 250), rather than the number of specimens used for molecular analysis.

For molecular genetic analyses, representative individuals were selected from the full collection to ensure broad coverage of the observed morphological variation and geographic distribution across sampling sites. Given the cryptic endophytic nature of *R. ferrugineus* within palm tissues and the logistical constraints associated with large-scale destructive sampling, this approach provides a practical and widely used framework for preliminary regional genetic assessment.

### 2.2. DNA Extraction

Genomic DNA was extracted from whole adult specimens to ensure sufficient yield of both mitochondrial and nuclear genetic material. Extraction was performed using the DNeasy Blood & Tissue Kit (Qiagen, Cat. No. 69506, Venlo, The Netherlands), following the manufacturer’s standardized protocol.

All extraction steps were conducted under sterile laboratory conditions to minimize contamination risk. Tissue lysis was performed using proteinase K digestion to ensure complete breakdown of chitinous insect tissues, followed by silica-membrane-based purification to obtain high-quality genomic DNA.

DNA concentration and purity were evaluated using a NanoDrop 1000 spectrophotometer (Thermo Scientific, Wilmington, DE, USA), measuring absorbance ratios at 260/280 nm to assess protein contamination. DNA integrity was further verified by electrophoresis on agarose gel, ensuring high molecular weight DNA suitable for PCR amplification. Only samples meeting quality thresholds were selected for downstream molecular analysis.

### 2.3. Molecular Markers and Rationale for Selection

#### 2.3.1. PCR Amplification

Two complementary molecular markers were selected for this study: COI and ITS2. PCR amplification of the COI gene was conducted following the protocol described by [2,22], which has been widely used for *R. ferrugineus* molecular characterization. The ITS region was amplified using the primer pair:

- ITS2-F: 5′-ATATGCTTAAATTCAGCGG-3′;
- ITS2-R: 5′-GGGTCGATGAAGAACGCAGC-3′ [7].

Each PCR reaction (50 µL total volume) contained:

- A total of 25 µL of 2× GoTaq Green Master Mix (Promega);
- A total of 20 pM of each forward and reverse primer;
- Approximately 40 ng of genomic DNA template;
- Nuclease-free water to complete final volume.

Thermal cycling was performed under the following conditions:

- Initial denaturation at 94 °C for 5 min to ensure complete DNA strand separation.
- 35 amplification cycles consisting of:
  ○ Denaturation at 94 °C for 30 s;
  ○ Primer annealing at 50 °C for 30 s;
  ○ Extension at 72 °C for 30 s.
- Final extension at 72 °C for 7 min to complete DNA synthesis.

PCR amplification success was confirmed using 1.5% agarose gel electrophoresis prepared in 1× TAE buffer. Electrophoresis was run at 100 V for 30 min. DNA bands were stained with ethidium bromide (0.5 µg/mL) and visualized under ultraviolet transillumination. A 100 bp DNA ladder (Thermo Fisher Scientific, Vilnius, Lithuania) was used to estimate fragment size. All genomic DNA samples were initially assessed for quality prior to PCR amplification, and no evidence of significant degradation was observed across the extracted samples.

#### 2.3.2. DNA Sequencing and Sequence Processing

Successful PCR products were purified and submitted for bidirectional Sanger sequencing using an ABI PRISM 3730XL Genetic Analyzer (Macrogen Inc., Seoul, Republic of Korea). Bidirectional sequencing was used to improve sequence accuracy and reduce base-calling errors.

Raw sequence chromatograms were carefully inspected to ensure sequence quality. Low-quality regions, ambiguous bases, and primer sequences were manually trimmed. Only sequences with clear and unambiguous bidirectional read support were included in the final dataset, and ambiguous nucleotide positions were excluded to minimize sequencing artefacts. Consensus sequences were generated by aligning forward and reverse reads, and only high-confidence sequences were retained for downstream analyses.

Sequence quality and assembly were performed using BioEdit software (version 7.7.1), allowing alignment of forward and reverse reads into consensus sequences. All sequences were then compared against the GenBank database using BLAST (MEGA X, version: 10.2.6) [35] to confirm species identity and verify sequence similarity with previously reported *Rhynchophorus* sequences.

#### 2.3.3. Sequence Alignment and Phylogenetic Reconstruction

Multiple sequence alignments were performed using ClustalW implemented in MEGA X software (version: 10.2.6) [35]. All alignments were carefully inspected and manually adjusted to minimize alignment artifacts and ensure positional homology across sequences. Following alignment, haplotypes were identified separately for COI and ITS datasets based on observed sequence variation. Phylogenetic relationships were reconstructed independently for COI and ITS datasets using the Maximum Likelihood (ML) method implemented in MEGA X. The Tamura–Nei substitution model was applied, as it accounts for unequal nucleotide frequencies and variable substitution rates among nucleotides [35].

The robustness of inferred phylogenetic relationships was evaluated using 10,000 bootstrap replicates to assess node stability and provide statistical support for tree topology. Bootstrap values were calculated for all nodes, and values ≥ 70% were considered to indicate strong support, whereas lower values were interpreted with caution in downstream interpretations [35]. Tree topology optimization was further refined using the Nearest-Neighbor Interchange (NNI) heuristic search algorithm to improve likelihood estimation through systematic evaluation of local rearrangements.

Phylogenetic interpretations were restricted to evolutionary relationships among sampled individuals and were not used to infer demographic processes such as gene flow or population structure, consistent with the analytical scope of distance- and tree-based methods [21,22,23,24].

#### 2.3.4. Sequence Submission to Genbank and Data Availability

From the total of 57 collected *R. ferrugineus* specimens, high-quality sequences were successfully obtained for 55 COI and 53 ITS datasets. All obtained sequences were submitted to the GenBank database to ensure public accessibility and support transparency and reproducibility of the molecular analyses conducted in this study (Table 1). The deposited sequences are available under the following accession numbers: COI (MW507794-MW507809; PP275884-PP275887; and PZ578201-PZ578235) and ITS (PP376129-PP376181). These publicly available datasets provide a genetic reference framework for future comparative and population-level studies of *R. ferrugineus* in the region.

#### 2.3.5. Sequence Variation and Genetic Diversity Analysis

The aligned nucleotide sequences for both the mitochondrial COI and nuclear ITS markers were analyzed to assess the genetic diversity and intraspecific variation in *R. ferrugineus* populations. For each marker, pairwise genetic distances (*p*-distance), the total number of sequences analyzed (*n*), the alignment length (*L*), the number of polymorphic sites (*S*), and the number of unique haplotypes (*h*) were determined from the aligned datasets from MEGA X software.

To quantify the genetic variation within the selected insects, haplotype diversity (*Hd*) and nucleotide diversity (π) were calculated. *Hd* was estimated using the unbiased formula proposed by Nei [36], representing the probability that two randomly chosen sequences are different haplotypes. Nucleotide diversity (π) was calculated as the average pairwise proportion of nucleotide differences between any two randomly chosen sequences in the sample.

To address the requirement for measures of statistical uncertainty, standard errors (SE) and 95% confidence intervals (CI) were computed for both diversity indices. The SE for *Hd* was estimated using a coalescent-based framework, while the SE for *π* was generated using a bootstrapping approach over sites with 10,000 replicates. The 95% CIs were subsequently derived from the bootstrap distributions to provide robust boundaries for the diversity estimates.

To statistically evaluate and compare the genetic variability between the two markers, the Z-score was calculated using the following equation:

Z = (π_ITS − π_COI)/√(SE^2^_ITS + SE^2^_COI)

where π_ITS and π_COI are the nucleotide diversity estimates, and SE_ITS and SE_COI are their corresponding standard errors for the ITS and COI markers, respectively. The calculated Z-score was evaluated against the standard normal distribution to determine the *p*-value, with statistical significance defined at *p* < 0.05 (DnaSP v6.0 [37]).

## 3. Results

### 3.1. Genetic Variation in Rhynchophorus Ferrugineus Populations

A total of 57 adult *R. ferrugineus* specimens collected from nine sampling locations in Qassim Province (SA1–SA9) were subjected to molecular characterization using COI and ITS markers. All specimens were processed for DNA extraction, PCR amplification, and sequencing for both loci. The sequencing success rates were 96.5% in COI and 93% with ITS2 markers, respectively, while two COI and four ITS samples failed to generate usable sequence data (Table 2).

The COI dataset comprised 55 valid sequences, including 16 previously available reference haplotypes (Accession numbers: MW507794–MW507809 [2]) and 39 newly generated sequences from the present study (Accession numbers: PP275884–PP275887 and PZ578201–PZ578235). Consequently, the newly generated sequences represented 70.91% of the total valid mitochondrial dataset, whereas previously reported sequences accounted for 29.09%. In contrast, the ITS dataset consisted of 53 newly generated sequences representing most of the collected specimens and deposited under accession numbers PP376129–PP376181, with no previously available ITS sequences included.

Overall, the high sequencing success rates and the substantial number of newly deposited sequences considerably expanded the molecular database available for the genetic characterization and population studies of *R. ferrugineus* populations in SA. Comparative interpretations between mitochondrial and nuclear markers were performed while considering differences in sequencing success rates and sample sizes between datasets.

Sequencing success rate (%) was calculated as the proportion of successfully sequenced samples relative to the total number of specimens analyzed. Contribution of newly generated sequences (%) was calculated as the proportion of newly deposited accession records relative to successfully sequenced samples.

#### 3.1.1. COI-Based Genetic Variation Among Qassim Specimens

Comparative analysis of mitochondrial COI sequences revealed consistently low levels of nucleotide divergence among the examined *R. ferrugineus* specimens from the Qassim region. Pairwise genetic distances (*p*-distance) ranged from 0.0 to 0.0077 across the analyzed haplotypes, corresponding to 0.0–0.77% sequence divergence, indicating a high degree of mitochondrial sequence conservation within the analyzed dataset (Table 3). Based on the observed genetic distance categories, most haplotypes belonged to highly conserved or low-variation groups (0.00–0.0058), whereas only a small subset exhibited moderate differentiation, reaching 0.0077. The overall mean pairwise divergence among Qassim haplotypes was low (0.0031), supporting the predominance of a conserved mitochondrial lineage with only limited internal diversification.

**Figure 4:**
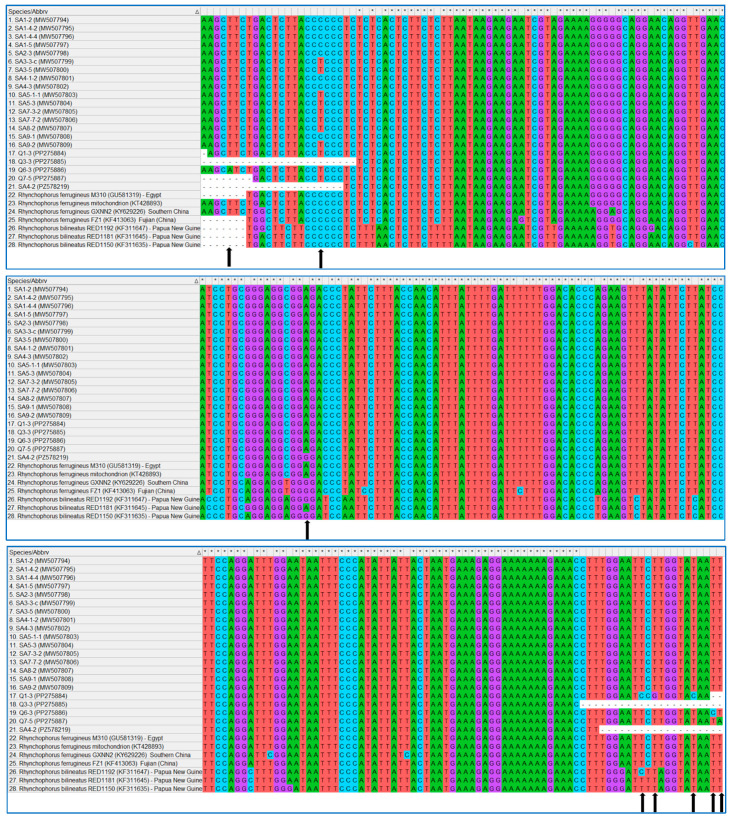
Alignment of COI sequences of *R. ferrugineus*. Arrows indicate nucleotide variations in the five newly identified haplotypes (Q1-3, Q3-3, Q6-3, and Q7-5 and SA4-2). * means the same nucleotide in all specimens.

This pattern reflects a low level of sequence differentiation at the COI locus among locally sampled individuals and suggests the existence of a relatively homogeneous mitochondrial background within the Qassim populations. Five additional COI haplotypes, designated Q1-3 (PP275884), Q3-3 (PP275885), Q6-3 (PP275886), Q7-5 (PP275887), and SA4-2 (PZ578219), were identified during the present study and exhibited sequence variation reaching up to 0.77%. Multiple sequence alignment using ClustalW in MEGA X detected minor nucleotide differences among these haplotypes relative to previously reported sequences. Specifically, Q1-3 differed by four nucleotide positions, Q6-3 by three nucleotide positions, Q7-5 by two nucleotide positions, and SA4-2 exhibited a single nucleotide substitution across the analyzed COI fragment (Figure 4).

The relatively small number of nucleotide substitutions observed among these haplotypes further supports the limited mitochondrial differentiation detected within the Qassim populations and is consistent with the overall pattern of weak internal genetic structuring revealed by the COI dataset.

#### 3.1.2. Mitochondrial COI-Based Phylogenetic Relationships and Genetic Structure of *R. ferrugineus* Specimens

**Figure 5:**
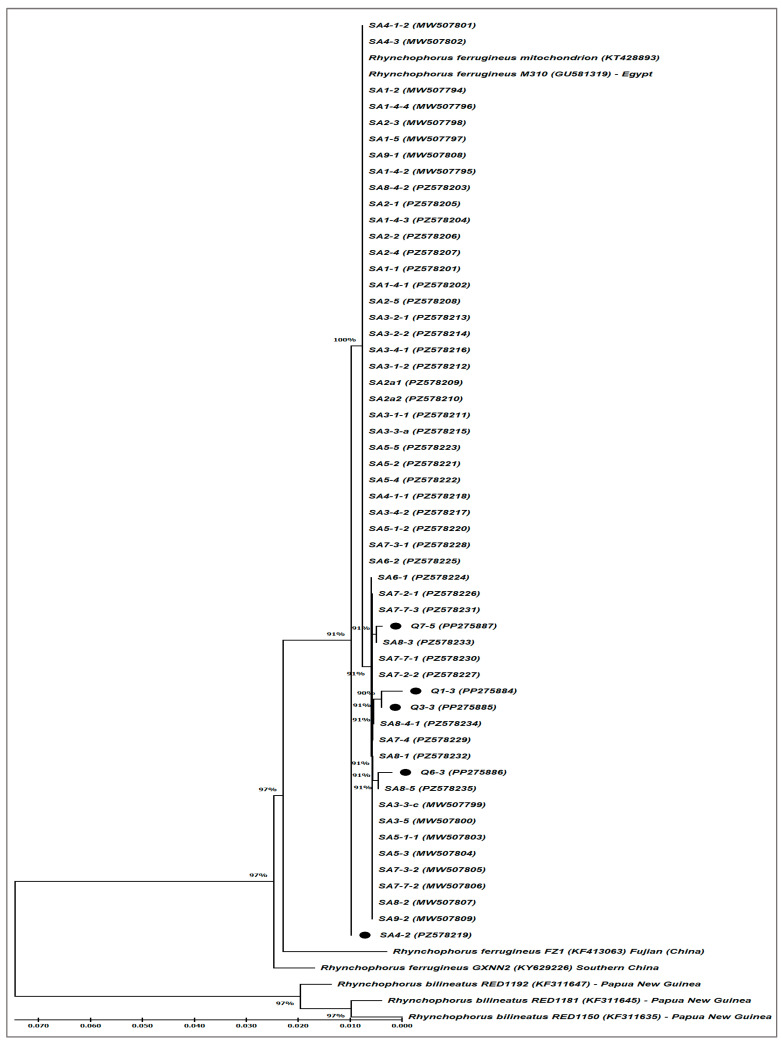
Maximum Likelihood phylogenetic tree based on mitochondrial COI sequences of *R. ferrugineus.* Phylogenetic relationships among COI sequences from Qassim (Saudi Arabia) and GenBank references were inferred using the Maximum Likelihood method under the Tamura–Nei model. Node support was evaluated with 10,000 bootstrap replicates. Newly generated 38 haplotypes such as Q1-3, Q3-3, Q6-3, Q7-5 and SA4-2 (●) are included. The scale bar indicates nucleotide substitutions per site.

Phylogenetic analysis based on mitochondrial COI sequences revealed a well-resolved clustering pattern among Qassim populations of *R. ferrugineus* and reference sequences (Table 4; Figure 5). The reconstructed phylogenetic tree resolved the analyzed sequences into three major clusters with several internal sub-clusters supported by moderate to high bootstrap values (90–100%), reflecting varying levels of genetic affinity among local, national, and international populations. Detailed pairwise nucleotide divergence values and comparative relationships among all analyzed haplotypes are provided in Supplementary File S1.

Cluster I represented the principal Saudi/Qassim lineage and included most Qassim haplotypes together with previously reported *R. ferrugineus* references (the Egyptian isolate (GU581319) and the mitochondrion reference (KT428893)), indicating high sequence similarity and close phylogenetic relationships among these mitochondrial lineages. Within this principal lineage, several internal sub-clusters were identified. The dominant haplotype group involved specimens such as SA1-2, SA4-3, SA9-1, SA2-1, SA1-4-3, SA2-2, SA3-1-1, SA5-1-2, and SA6-2, supporting the predominance of a common maternal lineage within the studied population. Minor sub-clustering was also observed among several derived haplotypes, where Q1-3 (PP275884) and Q3-3 (PP275885) clustered together, whereas Q7-5 (PP275887) and Q6-3 (PP275886) formed separate sub-groups with SA8-3 and SA8-5, respectively. In contrast, SA4-2 (PZ578219) occupied a relatively isolated position outside the principal Qassim sub-clusters and represented the most divergent haplotype within the Saudi population.

Comparisons among Qassim haplotypes and national reference haplotypes revealed limited sequence divergence and strong phylogenetic affinity, indicating a relatively conserved mitochondrial structure. Moreover, the clustering of Qassim sequences with the Egyptian isolate (GU581319) suggests possible regional connectivity and shared maternal ancestry among Middle Eastern populations. Pairwise comparisons between Saudi and geographically distant populations showed increasing levels of genetic divergence, with values ranging from approximately 0.0019 to 0.0446 (Supplementary File S1), indicating progressive differentiation associated with geographic distance.

Cluster II comprised Chinese *R. ferrugineus* sequences from Southern China (KY629226) and Fujian (KF413063), which formed a separate lineage outside the principal Qassim cluster with strong bootstrap support (97%), indicating greater regional differentiation among geographically distant populations.

Cluster III consisted of *R. bilineatus* reference sequences from Papua New Guinea and formed a distinct outgroup lineage with strong bootstrap support (97%), demonstrating clear species-level separation from all *R. ferrugineus* haplotypes. Comparison between Qassim haplotypes and *R. bilineatus* revealed substantially greater genetic divergence than that observed among *R. ferrugineus* populations. For example, the highest divergence value was detected between *R. bilineatus* RED1150 (KF311635) and Q1-3 (PP275884), with a *p*-distance value of 0.1413 (Supplementary File S1), indicating broad evolutionary separation between the two species.

Although no direct sister-group relationship was observed between Qassim haplotypes and *R. bilineatus*, SA4-2 (PZ578219) showed the closest topological position among Saudi haplotypes toward the branch leading to Chinese *R. ferrugineus* sequences and subsequently the *R. bilineatus* outgroup. However, the substantial branch separation indicates that this relationship reflects relative phylogenetic positioning rather than close evolutionary affinity. The COI dataset further indicated that most haplotypes differed by only a small number of nucleotide substitutions, reflecting the predominance of a common mitochondrial lineage with limited local diversification.

#### 3.1.3. ITS-Based Genetic Variation Among Qassim Specimens

**Figure 6:**
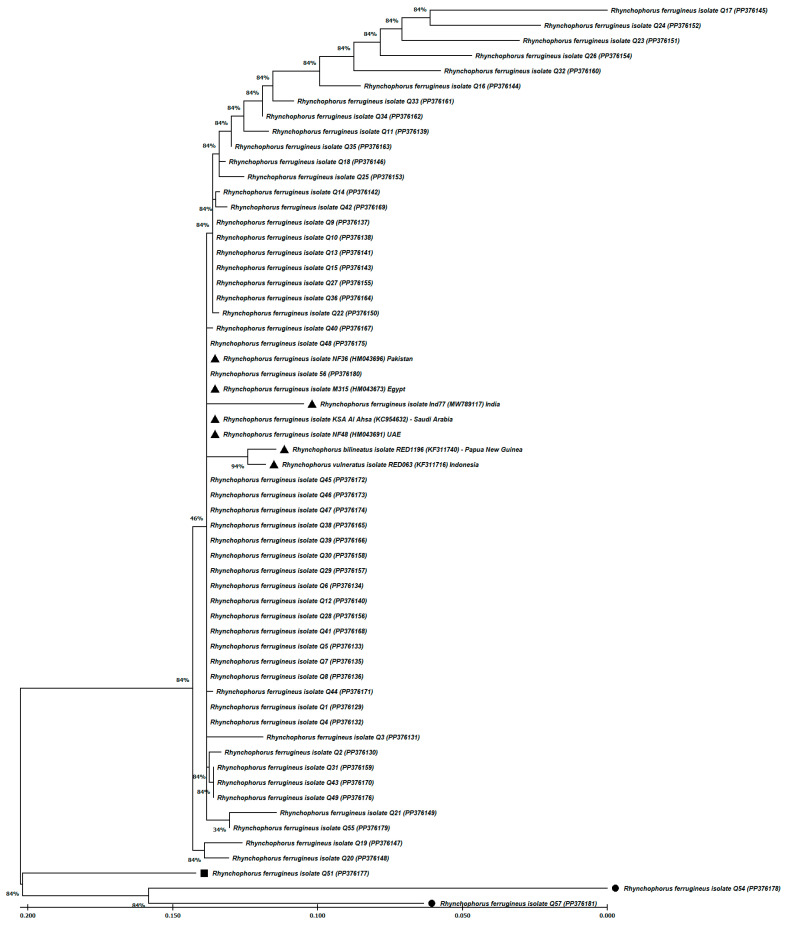
Maximum Likelihood phylogenetic tree based on nuclear ITS sequences of *R. ferrugineus.* Phylogenetic relationships among ITS sequences from Qassim and GenBank references were inferred using the Maximum Likelihood method under the Tamura–Nei model. Branch support was assessed using 10,000 bootstrap replicates. The scale bar represents nucleotide substitutions per site. ▲ point to reference haplotypes worldwide, ● and ■ refer to the most divergence haplotypes.

ITS sequence analysis was successfully completed for 53 specimens, revealing substantial sequence variation among the analyzed Qassim populations of *R. ferrugineus* (Table 5; Figure 6). Phylogenetic construction based on ITS sequences generated a complex clustering pattern among local and reference sequences, resolving the analyzed haplotypes into four major clusters with several internal sub-clusters. The observed phylogenetic structure reflected heterogeneous nuclear variation and differing levels of genetic association among local, national, and international populations. Detailed pairwise sequence relationships, genetic divergence values, and comparative analyses among local and reference ITS haplotypes are summarized in Table 5 and Supplementary File S2.

Cluster I represented the principal Qassim lineage and included the majority of local ITS haplotypes arranged into three major sub-clusters with bootstrap support of approximately 84%. The first sub-cluster comprised Q17, Q24, Q23, Q26, Q32, Q16, Q33, Q34, Q11, Q35, Q18, Q25, Q14, Q42, Q9, Q10, Q27, Q36, and Q22 (Table 5; Figure 6). The close association among these haplotypes indicates considerable genetic similarity and shared nuclear characteristics within a large proportion of the Qassim populations.

The second sub-cluster grouped several Qassim haplotypes such as Q40, Q48 and Q56 with national and international reference sequences from Saudi Arabia, Egypt, Pakistan, and India, indicating broader phylogenetic relationships among geographically separated populations. The clustering of several Qassim isolates with Egyptian, Pakistani, Indian, and Saudi reference sequences suggests possible regional connectivity and shared ancestry among these populations. The distribution of these haplotypes across the phylogenetic tree indicates varying degrees of sequence similarity among geographically distinct populations.

The 3rd sub-cluster contained the secondary Qassim lineage and contained numerous local isolates distributed across several internal branches, reflecting additional population structuring within the analyzed specimens. Most ITS haplotypes exhibited low to moderate levels of sequence divergence relative to one another, whereas a smaller subset of haplotypes showed comparatively greater variation. In addition, Q19 (PP376147) and Q20 (PP376148) displayed a distinctive sequence divergence pattern and formed an independent cluster (cluster II) within the phylogenetic tree. Similarly, Q51 (PP376177), Q54 (PP376178), and Q57 (PP376181) occupied relatively distinct phylogenetic positions and formed a separate grouping characterized by increased nucleotide variation (cluster III and IV).

Overall, the ITS dataset revealed broad phylogenetic structuring and heterogeneous sequence variation among the analyzed Qassim populations, indicating substantial nuclear diversity and local genetic differentiation within *R. ferrugineus*.

#### 3.1.4. Comparative Analysis of Local and Global COI Haplotypes

Comparative analysis between Qassim COI haplotypes and previously published regional and international sequences revealed generally low mitochondrial divergence among Middle Eastern populations of *R. ferrugineus* (Table 6; Supplementary File S1). The Egyptian reference haplotype (GU581319) and the reference *R. ferrugineus* mitochondrial genome (KT428893) exhibited exceptionally high sequence similarity to several Qassim-derived haplotypes (*p*-distance = 0.0–0.0078), differing by only a limited number of nucleotide substitutions and, in some cases, showing identical sequence profiles within the analyzed COI fragment.

In contrast, comparisons with geographically more distant populations demonstrated increasing levels of mitochondrial differentiation. Likewise, East Asian populations from Southern China and Fujian displayed increased sequence divergence relative to Qassim haplotypes, with pairwise genetic distances ranging from approximately 0.0233 to 0.0446, suggesting progressive mitochondrial differentiation associated with broader geographic separation.

Interspecific comparisons involving *R. bilineatus* references yielded substantially greater divergence values than those observed among *R. ferrugineus* populations, reaching approximately 0.1099–0.1413. These divergence levels substantially exceeded intraspecific variation and supported the clear evolutionary separation of these species from the analyzed *R. ferrugineus* populations, validating their suitability as external comparative taxa in phylogenetic analyses.

Overall, the COI-based analyses demonstrated limited mitochondrial divergence within Qassim populations and strong genetic affinity with previously reported Middle Eastern haplotypes, whereas increasing divergence levels were observed among geographically distant Asian populations and interspecific reference taxa.

#### 3.1.5. Comparative Analysis of ITS Sequence Variation Among Qassim and *R. ferrugineus* Reference Populations

Analysis of the ITS sequences identified multiple sequence variants (Q1–Q57), which are listed together with their corresponding GenBank accession numbers in Table 1. Comparative analyses were performed against representative *R. ferrugineus* ITS sequences originating from Egypt, Saudi Arabia (Al-Ahsa), Pakistan, the United Arab Emirates, and India (Table S3; Supplementary File S2).

Most Qassim ITS haplotypes exhibited relatively low nucleotide divergence when compared with Middle Eastern reference populations. Reference sequences from Egypt, Saudi Arabia (Al-Ahsa), Pakistan, and the United Arab Emirates showed very close phylogenetic relationships with several Qassim haplotypes, and multiple comparisons demonstrated identical or nearly identical sequence profiles within the analyzed ITS region. Pairwise nucleotide divergence for the majority of regional comparisons ranged from approximately 0.0000 to 0.0086, indicating a generally conserved nuclear background among geographically related *R. ferrugineus* populations.

Several Qassim haplotypes, including Q2, Q9, Q10, Q13, Q14, Q15, Q18, Q22, and Q27, exhibited low but measurable divergence relative to regional reference sequences, differing by only a limited number of nucleotide substitutions (Table S4). Although many ITS haplotypes were unique, most represented only minor sequence modifications within the principal *R. ferrugineus* lineage.

A smaller subset of Qassim ITS haplotypes demonstrated comparatively elevated divergence values relative to regional and international reference sequences. Haplotypes Q16, Q23, Q26, and Q32 showed moderate divergence levels ranging from approximately 0.0416 to 0.0699, whereas Q51 and Q57 exhibited substantially higher divergence values relative to several reference populations, with pairwise *p*-distance values reaching approximately 0.2423 (Figure 6; Table S5). These divergent haplotypes occupied relatively distinct phylogenetic positions within the reconstructed ITS tree and likely represent locally differentiated nuclear variants. Comparisons involving geographically more distant populations revealed increasing levels of sequence differentiation.

The Indian isolate Ind77 (MW789117) displayed comparatively greater divergence values ranging from approximately 0.0372 to 0.0462 depending on the analyzed Qassim haplotype, indicating increasing genetic separation relative to Middle Eastern populations. Overall, the ITS dataset revealed variation ranging from highly conserved regional lineages to comparatively differentiated local variants, indicating heterogeneous patterns of nuclear sequence diversity among Qassim *R. ferrugineus* populations.

#### 3.1.6. Comparative ITS Relationships Between Qassim *R. ferrugineus* Haplotypes and Related *Rhynchophorus* Species

Comparative analyses involving related *Rhynchophorus* species demonstrated broader sequence differentiation relative to intraspecific *R. ferrugineus* comparisons (Tables S6 and S7; Supplementary File S2). Several Qassim haplotypes exhibited relatively low to moderate pairwise divergence values relative to *R. bilineatus* and *R. vulneratus*. For many haplotypes, divergence values ranged from approximately 0.0239 to 0.0370, indicating relatively close ITS sequence similarity among these *Rhynchophorus* taxa within the analyzed ITS region.

Haplotypes Q1, Q4, Q5, Q6, Q7, Q8, and Q12–Q15 exhibited comparatively smaller divergence values relative to both *R. bilineatus* and *R. vulneratus* reference sequences. Phylogenetic analysis similarly showed that some Qassim haplotypes occupied nearby branching positions relative to these reference taxa within the ITS tree (Figure 6). Additional Qassim haplotypes, including Q3, Q11, Q16, Q19, Q20, Q21, Q23, Q25, Q26, Q32, Q33, and Q34, showed intermediate divergence values relative to *R. bilineatus* and *R. vulneratus*, generally ranging from approximately 0.0417 to 0.0961.

A smaller subset of haplotypes, particularly Q17, Q24, Q51, Q54, and Q57, exhibited comparatively higher divergence values relative to the two reference species, exceeding 10% in all comparisons (Table S7). Despite their differentiated phylogenetic positions, these haplotypes remained associated with the broader *R. ferrugineus* lineage and did not cluster directly with external *Rhynchophorus* species.

Collectively, these findings indicate that ITS variation within Qassim populations encompasses both highly conserved lineages and locally differentiated variants, while maintaining clear separation from related *Rhynchophorus* species. The observed pattern suggests greater nuclear heterogeneity and broader phylogenetic structuring among Qassim populations than would be expected from highly conserved sequence regions alone.

### 3.2. Comparative Evaluation of COI and ITS Datasets

Comparative evaluation of mitochondrial COI and nuclear ITS datasets revealed distinct patterns of genetic variation and phylogenetic resolution among the analyzed *R. ferrugineus* populations from Qassim Province (Table 7; Supplementary Files S1 and S2). Although both markers successfully characterized the analyzed specimens and demonstrated close relationships with previously reported regional populations, considerable differences were observed in their ability to detect sequence variation and population structuring.

The COI dataset showed relatively conserved mitochondrial variation, characterized by low sequence divergence among Qassim haplotypes and strong clustering with previously reported Middle Eastern populations including Saudi Arabia, Egypt, Pakistan, and the United Arab Emirates (Figure 5; Supplementary File S1). In contrast, the ITS dataset exhibited broader sequence variability and a more complex phylogenetic structure, reflected by greater internal branching patterns and a larger number of differentiated local variants (Figure 6; Supplementary File S2).

Comparative diversity indices further supported the contrasting genetic patterns observed between mitochondrial and nuclear markers (Table 7). Haplotype diversity was greater in the ITS dataset (Hd = 0.876 ± 0.041; 95% CI: 0.764–0.922) than in the COI dataset (Hd = 0.663 ± 0.068; 95% CI: 0.505–0.782), indicating greater haplotype variation within the nuclear marker. Likewise, nucleotide diversity was substantially higher in ITS (π = 0.0387 ± 0.0013; 95% CI: 0.036–0.041) compared with COI (π = 0.00105 ± 0.00025; 95% CI: 0.00056–0.00154), corresponding to an approximately 36.9-fold increase in nucleotide variation. These findings indicate substantially greater genetic variability and sequence differentiation within the ITS marker relative to COI.

Statistical comparison of nucleotide diversity estimates demonstrated that the observed differences between markers were highly significant (Z = 27.66, *p* < 0.001), confirming that the increased variability detected by ITS reflected true biological differences rather than sampling-associated variation. Consistent with these observations, the comparative marker assessment summarized in Table 7 indicated that ITS exhibited greater internal variation, broader phylogenetic subdivision, and stronger capacity for detecting local population differentiation, whereas COI showed greater sequence conservation and stronger phylogenetic stability.

Overall, the combined analyses demonstrated that both molecular markers provided complementary information for genetic characterization of *R. ferrugineus* populations. COI was more suitable for identifying stable mitochondrial lineages and broader phylogeographic relationships, whereas ITS provided higher discriminatory power for resolving local population structure and detecting fine-scale genetic variation among Qassim populations.

### 3.3. Phylogenetic Relationships and Clustering Patterns of Qassim R. ferrugineus Haplotypes

Most Qassim haplotypes clustered closely with previously reported *R. ferrugineus* sequences from regional populations in the Middle East, including Egypt, Saudi Arabia, Pakistan, and the United Arab Emirates, indicating generally low genetic differentiation among these populations. In both COI- and ITS-based phylogenies, several haplotypes showed close phylogenetic proximity to these regional reference sequences (Figure 5 and Figure 6).

A small subset of ITS haplotypes exhibited comparatively higher sequence divergence relative to *R. bilineatus* and *R. vulneratus* reference sequences (Table 6) and occupied closer phylogenetic positions to these taxa within the ITS phylogeny (Figure 6). However, ITS variation alone is not sufficient for species-level delimitation within *Rhynchophorus*, and these patterns are interpreted as intraspecific variation within the *R. ferrugineus* complex rather than evidence of distinct taxonomic entities. No intermediate clustering patterns or consistent signals suggesting interspecific admixture were observed in the present dataset. Comparisons with geographically distant populations from East Asia and South Asia showed higher genetic divergence than that observed among Middle Eastern populations (Table 4, Table 5 and Table 6), indicating increased sequence differentiation across broader geographic scales.

Overall, the combined COI and ITS analyses (Figure 5 and Figure 6; Table 2, Table 3, Table 4, Table 5 and Table 6) indicate close phylogenetic relationships among Middle Eastern *R. ferrugineus* populations, alongside moderate ITS-based variability within the Qassim samples.

### 3.4. Contrasting Mitochondrial and Nuclear Patterns of Genetic Diversity Within a Global Phylogenetic Framework

**Figure 7:**
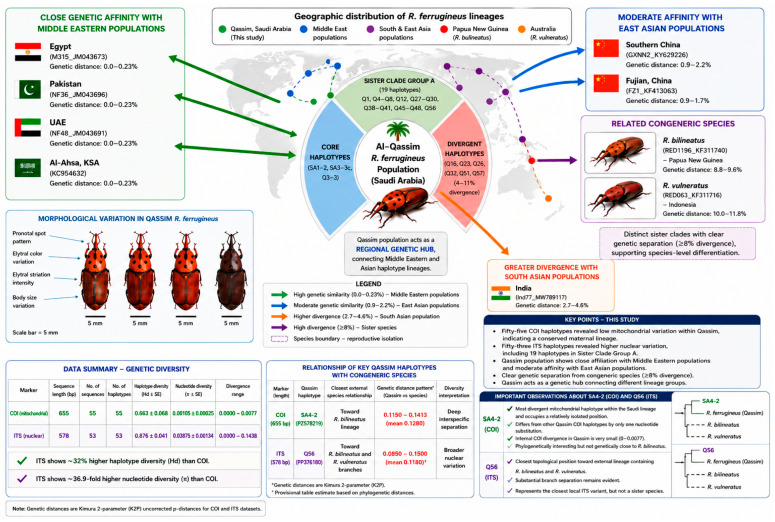
Contrasting mitochondrial and nuclear patterns of genetic diversity in Qassim *R. ferrugineus* populations within a global phylogenetic framework. Abbreviations: COI, mitochondrial cytochrome *c* oxidase subunit I gene; ITS, nuclear internal transcribed spacer region; *R. ferrugineus*. Blue indicates the mitochondrial COI dataset, whereas red indicates the nuclear ITS dataset, Dashed lines indicate inferred phylogenetic relationships among the mitochondrial (COI), nuclear (ITS), and global lineage datasets.

Figure 7 summarizes the global genetic relationships and phylogenetic placement of *R. ferrugineus* haplotypes from Qassim Province, inferred from the integrated interpretation of mitochondrial COI and nuclear ITS datasets. The comparative framework illustrates the contrasting patterns of sequence variation detected by the two molecular markers and highlights the position of Qassim populations within the broader *R. ferrugineus* species complex.

The majority of Qassim haplotypes occupied a central position within the global *R. ferrugineus* network and showed close genetic affinity with Middle Eastern populations, including Saudi Arabia, Egypt, Pakistan, and the United Arab Emirates. These populations formed a principal lineage characterized by very low sequence divergence and high genetic similarity, supporting the existence of a largely conserved regional genetic background. The strong phylogenetic connectivity among these populations suggests shared ancestry and possible regional movement of *R. ferrugineus* lineages.

A secondary assemblage comprised haplotypes showing moderate genetic differentiation and closer phylogenetic relationships with geographically distant Asian populations, including Southern China, Fujian (China), and India. The observed divergence likely reflects historical dispersal processes, lineage separation, or regional evolutionary differentiation among worldwide populations.

Additional local variants occupied relatively distinct positions within the genetic framework and represented divergent Qassim haplotypes, exhibiting increased sequence variation. These divergent forms were particularly evident within the nuclear ITS dataset and contributed to the broader internal structuring observed among local populations. Their distribution suggests the existence of heterogeneous evolutionary histories and localized differentiation within the Qassim population.

Comparisons with related *Rhynchophorus* species further demonstrated clear species-level separation. *R. bilineatus* and *R. vulneratus* formed distinct external lineages separated by broader genetic distances relative to intraspecific *R. ferrugineus* comparisons. Among the analyzed Qassim variants, Q56 occupied the nearest topological position toward the external *Rhynchophorus* lineage, representing the closest phylogenetic placement among local haplotypes. However, considerable branch separation remained evident, indicating relative phylogenetic proximity rather than close evolutionary relatedness. Collectively, the integrated phylogenetic framework highlights Qassim populations as an important regional genetic node linking Middle Eastern and Asian lineages while preserving overall species integrity within *R. ferrugineus*. The contrasting patterns observed between mitochondrial COI and nuclear ITS markers further demonstrate the complementary nature of these datasets, where mitochondrial sequences reflected conserved lineage relationships, whereas nuclear ITS markers provided greater resolution for detecting local genetic variation and population structuring.

## 4. Discussion

### 4.1. Comparative Assessment of Mitochondrial and Nuclear Markers in R. ferrugineus Populations from Qassim Province

The present study provides a regional-scale comparative assessment of mitochondrial (COI) and nuclear (ITS) genetic variation in *R. ferrugineus* populations from Qassim Province, Saudi Arabia, with particular emphasis on contrasting diversity patterns revealed by both molecular systems. A total of 57 specimens collected from geographically distinct localities were analyzed using both markers, resulting in high sequencing success rates for COI (96.5%) and ITS (93%) [26,27,28,29,30]. The incorporation of newly generated molecular data substantially expanded the available genetic dataset for Saudi Arabian *R. ferrugineus* populations and provided an opportunity to directly compare the performance of mitochondrial and nuclear markers in detecting population-level variation [2,22].

The comparative approach adopted in this study revealed clear differences in the genetic signals obtained from both markers. Although both molecular systems successfully characterized the analyzed specimens, they differed considerably in their ability to detect genetic variation and resolve phylogenetic relationships. The contrasting patterns observed between COI and ITS suggest that different molecular systems may capture different components of evolutionary history and population diversity. Such differences likely reflect fundamental biological distinctions between mitochondrial and nuclear genomes, including inheritance mechanisms, mutation dynamics, and evolutionary constraints [21,22,23,24,38,39].

The present analyses were designed primarily to evaluate detectable sequence variation and comparative marker performance rather than to estimate population-wide demographic parameters or conduct genome-scale population inference. Accordingly, the observed diversity indices and haplotype distributions should be interpreted as comparative measures within the analyzed dataset rather than absolute estimates of regional population frequencies. Nevertheless, the sampling strategy incorporated specimens representing broad geographic and observable phenotypic variation across Qassim Province, providing valuable baseline information regarding molecular diversity patterns in local *R. ferrugineus* populations [21,22,23,24,40,41,42,43,44,45,46,47,48,49,50].

Differences in sequencing success between markers were relatively limited and likely reflected variations in sequence quality and locus-specific amplification characteristics rather than biological differences among specimens. Such variation is common in comparative molecular studies and does not alter the overall interpretation of contrasting diversity patterns observed between mitochondrial and nuclear datasets.

### 4.2. Contrasting Genetic Signals Revealed by Mitochondrial and Nuclear Markers

A major finding of the present study was the clear contrast in genetic diversity patterns detected by mitochondrial and nuclear datasets. Although both molecular systems successfully characterized *R. ferrugineus* specimens from Qassim Province, they differed markedly in their capacity to detect sequence variation and resolve internal genetic relationships. The COI dataset exhibited relatively low nucleotide diversity and a highly conserved haplotype structure among the analyzed specimens, indicating a comparatively homogeneous mitochondrial background within the studied populations. Such patterns have frequently been associated with invasive insect populations and may reflect historical demographic processes such as founder effects, population expansion, or repeated introductions of closely related maternal lineages [21,22,23,24,40,41,42,43,44,45,46,47,48,49,50].

In contrast, the ITS dataset revealed substantially greater nuclear variation and more complex internal phylogenetic structuring. The broader range of sequence divergence, increased haplotype diversity, and greater branching complexity observed within the ITS phylogeny suggest that nuclear markers may capture evolutionary signals and local genetic differentiation that remain undetected in mitochondrial datasets. Similar differences between mitochondrial and nuclear markers have been reported in comparative molecular studies, where nuclear loci provided improved resolution of closely related lineages and intraspecific variation [22,23,24,25,26].

The higher discriminatory capacity observed in the ITS dataset may be attributed partly to biological differences between mitochondrial and nuclear genomes. Unlike maternally inherited mitochondrial DNA, nuclear markers undergo biparental inheritance and recombination, potentially retaining a broader spectrum of genetic information across populations [23,24,25,26]. The significantly greater haplotype and nucleotide diversity observed in ITS therefore supports the interpretation that nuclear markers may provide enhanced sensitivity for detecting fine-scale variation within *R. ferrugineus* populations.

Nevertheless, interpretation of ITS variation requires careful consideration because the ITS region represents a multicopy nuclear marker. Previous studies have indicated that ITS diversity can sometimes be influenced by incomplete concerted evolution, intra-genomic sequence heterogeneity, or amplification of paralogous sequence copies [27,28]. However, despite these recognized limitations, the observed ITS patterns in the present study remained consistent with broader phylogenetic relationships and revealed additional diversity signals not captured by mitochondrial data alone.

Differences in sequencing success between markers were relatively small, with COI generating usable sequences for 55 specimens (96.5%) and ITS for 53 specimens (93%). Therefore, the contrasting genetic patterns observed between datasets are unlikely to reflect methodological artifacts alone and instead appear to represent biologically meaningful differences in the evolutionary information captured by mitochondrial and nuclear marker systems [2,22,23,24,25,26].

### 4.3. Phylogenetic Relationships and Regional Genetic Connectivity of R. ferrugineus

Phylogenetic reconstruction based on both mitochondrial COI and nuclear ITS markers consistently assigned all analyzed haplotypes to *R. ferrugineus*, supporting species-level cohesion among the Qassim populations. Nevertheless, both molecular systems revealed contrasting patterns of internal genetic organization. COI phylogeny resolved a relatively conserved genetic structure characterized by a dominant mitochondrial lineage with limited internal divergence, whereas ITS analyses revealed broader phylogenetic branching and greater differentiation among local haplotypes. These contrasting topologies further support the interpretation that mitochondrial and nuclear markers capture different components of evolutionary history and genetic diversity [2,22,23,24,25,26].

Comparative analyses demonstrated close genetic relationships between Qassim haplotypes and previously reported *R. ferrugineus* populations from Saudi Arabia, Egypt, Pakistan, and the United Arab Emirates, indicating substantial genetic similarity among Middle Eastern populations [1,2,3,18,19,20]. The relatively limited divergence observed among these populations suggests the existence of a shared regional genetic background, potentially maintained through historical movement of infested planting materials and anthropogenic dispersal pathways that have been recognized as major drivers of red palm weevil expansion [21,22,23,24]. In contrast, geographically distant populations, particularly those from China and India, exhibited comparatively greater sequence divergence, supporting increased genetic differentiation associated with broader geographic separation and independent evolutionary trajectories [20,21,44,45,46,47].

The present analyses also identified a subset of ITS haplotypes exhibiting comparatively elevated sequence divergence relative to other local variants. In particular, haplotypes such as Q51, Q54, and Q57 occupied relatively distinct phylogenetic positions within the nuclear dataset, suggesting the presence of additional local genetic variation within Qassim populations. Although the origin of these divergent variants remains uncertain, similar patterns have been reported for multicopy ITS regions where sequence heterogeneity may arise from complex evolutionary processes, including incomplete concerted evolution and intra-genomic variation [27,28]. Additional nuclear loci and genome-wide analyses would, therefore, be valuable for clarifying the biological significance of these divergent sequence patterns.

An additional observation was the relatively closer phylogenetic placement of some local haplotypes toward related *Rhynchophorus* taxa. Among the analyzed specimens, haplotype Q56 occupied the nearest topological position toward branches containing *R. bilineatus* and *R. vulneratus*. However, all analyzed specimens remained firmly nested within the principal *R. ferrugineus* lineage and retained clear phylogenetic separation from external taxa. This pattern likely reflects shared evolutionary signals among closely related species within the genus rather than evidence of direct evolutionary affinity or species boundary overlap [23,24,25,26,27,28,29,30,41].

Overall, the phylogenetic findings highlight the presence of structured genetic variation within Qassim populations while simultaneously supporting the broader conclusion that combining mitochondrial and nuclear datasets provides a more comprehensive understanding of evolutionary relationships and regional genetic connectivity in *R. ferrugineus*.

### 4.4. Biological Significance and Implications of Contrasting Mitochondrial and Nuclear Diversity Patterns

The contrasting patterns of genetic diversity observed between mitochondrial and nuclear datasets provide important insights into the genetic architecture of *R. ferrugineus* populations in Qassim Province. While the mitochondrial dataset suggested the predominance of a relatively conserved regional lineage, the nuclear ITS dataset revealed broader genetic variability and additional internal differentiation among local haplotypes. The coexistence of conserved mitochondrial backgrounds and comparatively variable nuclear lineages indicates that different components of the genome may retain distinct evolutionary signals and reflect different aspects of population history.

Differences in the ability of mitochondrial and nuclear markers to resolve genetic variation have been reported across multiple insect groups [24,25,26,27,28]. Mitochondrial genes such as COI have been extensively used in phylogeographic and DNA barcoding studies because of their maternal inheritance and relatively conserved evolutionary characteristics. However, mitochondrial loci may provide limited sensitivity for detecting recent diversification events and fine-scale population differentiation [24,25,26]. Conversely, nuclear markers such as ITS frequently exhibit higher sequence variability and may retain additional information associated with local diversification and evolutionary processes [25,26]. The present findings support these observations and demonstrate that nuclear datasets may uncover patterns of diversity that remain unresolved in mitochondrial analyses alone.

The greater diversity detected by ITS in the present study is consistent with previous studies reporting improved resolution of population structure and intraspecific variation using nuclear markers [22,23,24,25,26,27,28]. Integrative molecular approaches combining mitochondrial and nuclear datasets have increasingly been recognized as providing stronger phylogenetic resolution and more reliable characterization of genetic relationships than reliance on single-marker systems [23,26,27,28].

The observed genetic structure among Qassim populations likely reflects a combination of regional connectivity and localized diversification processes. The close genetic association between many Qassim haplotypes and Middle Eastern populations supports the existence of a shared regional genetic background, potentially influenced by anthropogenic movement of infested plant materials and long-term dispersal pathways recognized in *R. ferrugineus* invasion history [1,2,3,4,5,6]. Simultaneously, the occurrence of more divergent nuclear variants indicates the presence of additional local variation that may reflect ongoing microevolutionary processes within established populations.

From an evolutionary perspective, maintenance of genetic variability within invasive populations may contribute to adaptive potential and long-term population persistence under changing environmental conditions. Genetic variation can influence biological traits associated with dispersal capability, host utilization, physiological responses, and adaptation to environmental stressors [1,39,44]. Although such mechanisms were not directly examined in the present study, the detected diversity patterns suggest that genetic variation may represent an important component influencing the ecological success and persistence of *R. ferrugineus* populations.

From an applied perspective, the present findings further emphasize the value of integrating molecular information into surveillance and Integrated Pest Management (IPM) frameworks. Understanding patterns of genetic variation and lineage relationships may contribute to improved monitoring programs, facilitate early detection of potential introductions, and strengthen molecular tracking of pest movement across regions [45,46,49]. Because population heterogeneity can potentially influence biological characteristics and responses to management strategies, consideration of genetic variation may improve future decision-making associated with control programs [1,4,5,6,22,51,52].

More importantly, the present findings demonstrate that mitochondrial and nuclear markers do not necessarily provide identical evolutionary signals, but instead reveal complementary dimensions of population diversity and genetic structure [53,54,55]. While mitochondrial COI efficiently resolved species identity and broader phylogeographic relationships, the ITS marker detected additional genetic variation and finer-scale patterns of local differentiation that remained unresolved in mitochondrial analyses [41,56]. These contrasting genetic signatures suggest that reliance on a single molecular marker may provide an incomplete representation of the underlying genetic complexity within invasive pest populations [55,56].

The molecular baseline established in the present study provides an important framework for future investigations of red palm weevil populations within Saudi Arabia and across broader invaded regions. Future studies should expand geographic sampling and integrate additional high-resolution genomic approaches, including single nucleotide polymorphism (SNP)-based analyses, whole mitochondrial genome sequencing, and population genomic datasets to further clarify invasion pathways, evolutionary dynamics, and adaptive mechanisms [54,55]. Integrating genomic, ecological, and environmental datasets may substantially improve our understanding of population establishment and dispersal processes, ultimately contributing to more accurate molecular surveillance systems and supporting the development of sustainable and genetically informed management strategies for *R. ferrugineus* [41,54,55,56,57].

These findings not only strengthen the current understanding of the evolutionary biology of this globally important pest but also provide a valuable genetic baseline for improving molecular diagnostics, population monitoring, and the development of genetically informed management strategies [41,54]. Future investigations should expand spatial and temporal sampling and incorporate high-resolution genomic approaches, including SNP-based analyses, whole mitochondrial genome sequencing, and population genomic datasets integrated with ecological and environmental variables, to further resolve invasion pathways, adaptive responses, and mechanisms underlying population establishment and spread [52,57]. Such integrative approaches may contribute to the development of predictive surveillance systems and more sustainable long-term management programs for *R. ferrugineus* across invaded regions.

## 5. Conclusions

The findings of the present study demonstrate that comparative analyses of mitochondrial COI and nuclear ITS markers revealed contrasting yet complementary patterns of genetic diversity within *R. ferrugineus* populations from Qassim Province, Saudi Arabia. COI analyses revealed relatively low nucleotide divergence and limited haplotypic differentiation, suggesting a highly conserved mitochondrial background among the analyzed specimens. In contrast, ITS analyses detected greater sequence variation, broader haplotype diversity, and more complex phylogenetic structuring, indicating higher levels of nuclear genetic variation and stronger population differentiation.

The observed differences between mitochondrial and nuclear datasets emphasize the complementary value of integrating multiple molecular markers in population genetic studies. While COI remained effective for species identification and broader phylogeographic relationships, ITS provided improved resolution of local genetic structure and additional diversity patterns not fully resolved by mitochondrial analyses. The combined use of both marker systems therefore enhanced the overall characterization of genetic variation and population relationships within the analyzed populations.

The study is limited by a relatively restricted and non-random sampling design and by the use of only two genetic markers, limiting broader genome-wide inference. Therefore, diversity estimates should be interpreted within the context of the analyzed dataset and not generalized directly to larger regional populations. Future studies incorporating expanded geographic sampling, genome-wide markers (e.g., SNP-based approaches), and integrated mitochondrial–nuclear datasets are recommended to provide deeper insights into invasion pathways, population dynamics, and evolutionary processes in *R. ferrugineus.* Collectively, the present findings establish an important molecular baseline for future genetic monitoring and genetically informed management strategies for this economically important invasive pest.

## Figures and Tables

**Table 1:** Accession numbers of *R. ferrugineus* samples from Qassim, Saudi Arabia, analyzed using COI and ITS markers.

Specimen No.	Specimen Code (COI)	Specimen Code (ITS)	COI		ITS Accession No.
			Accession No.	Status	
1	SA1-1	Q1	PZ578201	New	PP376129
2	SA1-2	Q2	MW507794	Reference # 2	PP376130
3	Q1-3	Q3	PP275884	New	PP376131
4	SA1-4-1	Q4	PZ578202	New	PP376132
5	SA1-4-2	Q5	MW507795	Reference # 2	PP376133
6	SA1-4-3	Q6	PZ578204	New	PP376134
7	SA1-4-4	Q7	MW507796	Reference # 2	PP376135
8	SA1-5	Q8	MW507797	Reference # 2	PP376136
9	SA2-1	Q9	PZ578205	New	PP376137
10	SA2-2	Q10	PZ578206	New	PP376138
11	SA2-3	Q11	MW507798	Reference # 2	PP376139
12	SA2-4	Q12	PZ578207	New	PP376140
13	SA2-5	Q13	PZ578208	New	PP376141
14	SA2a1	Q14	PZ578209	New	PP376142
15	SA2a2	Q15	PZ578210	New	PP376143
16	SA3-1-1	Q16	PZ578211	New	PP376144
17	SA3-1-2	Q17	PZ578212	New	PP376145
18	SA3-2-1	Q18	PZ578213	New	PP376146
19	SA3-2-2	Q19	PZ578214	New	PP376147
20	Q3-3	Q20	PP275885	New	PP376148
21	SA3-3-a	Q21	PZ578215	New	PP376149
22	SA3-3-b	Q22	—	—	PP376150
23	SA3-3-c	Q23	MW507799	Reference # 2	PP376151
24	SA3-4-1	Q24	PZ578216	New	PP376152
25	SA3-4-2	Q25	PZ578217	New	PP376153
26	SA3-5	Q26	MW507800	Reference # 2	PP376154
27	SA3-6	Q27	—	—	PP376155
28	SA4-1-1	Q28	PZ578218	New	PP376156
29	SA4-1-2	Q29	MW507801	Reference # 2	PP376157
30	SA4-2	Q30	PZ578219	New	PP376158
31	SA4-3	Q31	MW507802	Reference # 2	PP376159
32	SA5-1-1	Q32	MW507803	Reference # 2	PP376160
33	SA5-1-2	Q33	PZ578220	New	PP376161
34	SA5-2	Q34	PZ578221	New	PP376162
35	SA5-3	Q35	MW507804	Reference # 2	PP376163
36	SA5-4	Q36	PZ578222	New	PP376164
37	SA5-5	Q37	PZ578223	New	—
38	SA6-1	Q38	PZ578224	New	PP376165
39	SA6-2	Q39	PZ578225	New	PP376166
40	Q6-3	Q40	PP275886	New	PP376167
41	SA7-2-1	Q41	PZ578226	New	PP376168
42	SA7-2-2	Q42	PZ578227	New	PP376169
43	SA7-3-1	Q43	PZ578228	New	PP376170
44	SA7-3-2	Q44	MW507805	Reference # 2	PP376171
45	SA7-4	Q45	PZ578229	New	PP376172
46	Q7-5	Q46	PP275887	New	PP376173
47	SA7-7-1	Q47	PZ578230	New	PP376174
48	SA7-7-2	Q48	MW507806	Reference # 2	PP376175
49	SA7-7-3	Q49	PZ578231	New	PP376176
50	SA8-1	Q50	PZ578232	New	—
51	SA8-2	Q51	MW507807	Reference # 2	PP376177
52	SA8-3	Q52	PZ578233	New	—
53	SA8-4-1	Q53	PZ578234	New	—
54	SA8-4-2	Q54	PZ578203	New	PP376178
55	SA8-5	Q55	PZ578235	New	PP376179
56	SA9-1	Q56	MW507808	Reference # 2	PP376180
57	SA9-2	Q57	MW507809	Reference # 2	PP376181

**Table 2:** Summary statistics and contributions of COI and ITS sequence datasets generated for *R. ferrugineus* populations from Qassim, Saudi Arabia.

Parameter	COI Marker	ITS Marker
Total specimens analyzed	57	57
Successfully sequenced samples	55	53
Sequencing success rate (%)	96.5	93
Missing sequences	2	4
Newly generated sequences	39	53
Previously available reference sequences	16	0
Contribution of newly generated sequences (%)	70.19	100.00
Reference sequence proportion (%)	29.09	0.00

**Table 3:** Genetic variation among Qassim (Saudi Arabia) *Rhynchophorus ferrugineus* haplotypes based on pairwise nucleotide divergence (*p*-distance) of the COI markers.

Genetic Variation Category	Genetic Distance Range (*p*-Distance) ^1^	Mean *p*-Distance ^2^	Biological Interpretation ^3^
Identical or highly conserved haplotypes	0.0–0.0021	0.0005	Represents dominant mitochondrial lineages with identical or nearly identical sequences
Low variation haplotypes	0.0021–0.0058	0.0038	Indicates minor sequence diversification among local haplotypes
Moderately differentiated haplotypes	0.0058–0.0077	0.0067	Reflects limited local mitochondrial differentiation
Overall within-Qassim variation	0.0–0.0077	0.0031	Supports low overall mitochondrial diversity and weak internal population structuring

^1^ Genetic distance range (*p*-distance): Proportion of nucleotide differences observed among compared COI haplotypes. ^2^ Mean *p*-distance: Average pairwise nucleotide divergence within each category. ^3^ Biological interpretation: Evolutionary interpretation of the observed sequence variation and its implications for population diversity.

**Table 4:** Phylogenetic clustering pattern of *R. ferrugineus* COI haplotypes from Qassim populations and reference sequences.

Major cluster	Sub-Cluster	Representative Sequences	Bootstrap Support (%)	Phylogenetic Interpretation
Cluster I: Saudi/Qassim lineage	I-A	Most of identified specimens such as SA1-2, SA4-3, SA9-1, SA2-1, SA1-4-3, SA2-2, SA3-1-1, SA5-1-2, and SA6-2 together with Egypt reference (GU581319) and mitochondrial reference (KT428893).	100	Dominant mitochondrial haplotype with highly conserved COI sequences
	I-B	Q7-5 (PP275887), Q1-3 (PP275884), Q3-3 (PP275885), Q6-3 (PP275886) and neighboring specimens	90–91	Minor derived haplotypes differing by few nucleotide substitutions
	I-D	SA4-2 (PZ578219)	91	Most divergent haplotype within the Saudi lineage
Cluster II: Asian lineage	II-A	*R. ferrugineus* GXNN2 (KY629226), Southern China; *R. ferrugineus* FZ1 (KF413063), Fujian, China	97	Distinct geographic lineage indicating regional differentiation
Cluster III: Outgroup lineage	III-A	*Rhynchophorus bilineatus* RED1192, RED1181, RED1150 (Papua New Guinea)	97	Outgroup cluster showing species-level divergence

Cluster designation was based on COI phylogenetic topology and branch support values generated from the reconstructed phylogenetic tree. Bootstrap values indicate the confidence level for major branches.

**Table 5:** Phylogenetic clustering pattern of *R. ferrugineus* ITS haplotypes from Qassim populations and reference sequences.

Major Cluster	Sub-Cluster	Representative Sequences	Bootstrap Support (%)	Phylogenetic Interpretation
Cluster I	I-A	Main Qassim lineage: Q17, Q24, Q23, Q26, Q32, Q16, Q33, Q34, Q11, Q35, Q18, Q25, Q14, Q42, Q9, Q10, Q13, Q15, Q27, Q36, and Q22	~84	Dominant Qassim lineage with showing closely related or moderate sequence variation
	I-B	International-reference lineage: Pakistan (NF36), Egypt (M315), India (Ind77), Saudi reference (KSA Al Ahsa), and UAE (NF48), in addition to local specimens such as Q40, Q48, and Q56.	84–94	National and international reference haplotypes clustered with selected Qassim sequences
	I-C	Secondary Qassim lineage: Q45, Q46, Q47, Q38, Q39, Q30, Q29, Q6, Q12, Q28, Q41, Q5, Q7, Q8, Q44, Q1, Q4, Q3, Q2, Q31, Q43, Q49, Q21, and Q55	34–84	Large secondary Qassim assemblage indicating moderate diversity
Cluster II	II-A	Q19, Q20	84	Haplotypes with increased local diversity
Cluster III: Divergent ITS haplotypes	III-A	Q51	84	Distinct lineage separated from major Qassim groups
Cluster IV: Divergent ITS haplotypes	IV-A	Q54 and Q57	—	Highly divergent local haplotype

**Table 6:** Integrated comparative analysis of Qassim and regional/global *Rhynchophorus ferrugineus* COI haplotypes based on phylogenetic relationships and genetic divergence patterns.

Comparative Group ^1^	Representative Populations/Accessions ^2^	Genetic Distance Range (*p*-Distance) ^3^	Mean ^4^	Genetic Identity (%) ^5^	Interpretation ^6^
Egypt/type mitochondrion	GU581319- KT428893	0.0–0.0078	0.0032	99.55–100.00	Nearly identical mitochondrial lineage with strong regional affinity
Mixed Qassim haplotypes	SA1–SA9 populations	0.0021–0.0077	0.0045	99.14–99.79	Minor internal differentiation among local haplotypes
China haplotypes	KY629226–KF413063	0.0233–0.0446	0.0314	96.31- 97.41	Moderate genetic diversity within the same species
*R. bilineatus*	RED1150, RED1181, RED1192	0.1099–0.1413	0.1300	85.87–88.00	Clear species-level separation
Qassim haplotypes vs. both species	SA-series vs. *R. bilineatus*/*R. vulneratus*	0.1150–0.1413	0.1280	85.87–88.50	Stable interspecific divergence
High divergence (>10%)	*R. bilineatus* and *R. vulneratus* comparisons	0.1150–0.1413	0.1280	85.87–88.50	Deep phylogenetic separation
Extreme divergence	Maximum interspecific comparisons	>0.1413	0.1413	<85.87	Broad evolutionary separation

^1^ Comparative group: Population, species, or divergence category compared with Qassim COI haplotypes. ^2^ Representative populations/accessions: GenBank accession numbers or representative isolates included in each category. ^3^ Genetic distance range (*p*-distance): Minimum–maximum pairwise nucleotide divergence among aligned COI sequences. ^4^ Mean: Average pairwise nucleotide divergence for each comparative category. ^5^ Genetic identity (%): Sequence similarity estimated was as Genetic identity=(1−p-distance)×100. ^6^ Interpretation: Biological meaning of the observed genetic divergence patterns.

**Table 7:** Comparative evaluation of mitochondrial COI and nuclear ITS datasets for genetic characterization of *Rhynchophorus ferrugineus* populations from Qassim, Saudi Arabia.

Parameter	COI Dataset	ITS Dataset	Comparative Interpretation
Marker type	Mitochondrial DNA	Nuclear ribosomal DNA	Represents maternal inheritance versus biparental nuclear inheritance
Molecular region	Cytochrome c oxidase subunit I	Internal transcribed spacer	Different evolutionary rates and inheritance patterns
Total specimens analyzed	57	57	Same biological samples
Successfully sequenced samples	55	53	COI showed slightly higher sequencing efficiency
Sequencing success (%)	96.5%	93%	COI amplified more consistently
Number of failed sequences	2	4	Greater sequence loss in ITS
Number of analyzed haplotypes	55 COI haplotypes	53 ITS haplotypes	Similar overall dataset sizes
Number of newly generated sequences	39	53	ITS dataset entirely generated in this study
Percentage of newly generated sequences	70.91%	100.00%	ITS contributed a larger novel sequence resource
Sequence conservation	High	Moderate	COI more conserved than ITS
Internal nucleotide variation	Low	Moderate–high	Greater heterogeneity in ITS
Pairwise divergence among local haplotypes	0.0–0.0077	0.0–0.2599	ITS detected broader variation
Mean divergence within Qassim populations	Very low	Moderate	Greater population differentiation in ITS
Principal phylogenetic clusters	3	4	ITS resolved more major groups
Number of sub-clusters	5	Multiple (>6)	Greater internal branching in ITS
Bootstrap support	90–100%	34–94%	COI exhibited stronger node support
Dominant lineage pattern	One principal mitochondrial lineage	Multiple nuclear lineages	ITS revealed more complex structure
Relationship with Middle East populations	Very close	Very close	Both markers indicated regional connectivity
Relationship with India populations	Moderate divergence	Moderate divergence	Geographic differentiation detected by both
Relationship with Chinese populations	Moderate divergence	Not represented	Only evaluated in COI
Relationship with *R. bilineatus*	Strong species separation	Strong species separation	Both markers resolved species boundaries
Most divergent local variants	SA4-2	Q51, Q54, Q57	ITS detected more divergent local variants
Geographic discrimination capacity	Moderate	High	ITS showed better geographic resolution
Population differentiation capacity	Moderate	High	ITS better resolved internal diversity
Ability to detect local structuring	Limited	Strong	ITS identified broader subdivision patterns
Suitability for species identification	Excellent	Good–excellent	COI remains more stable for species identification
Suitability for population genetic studies	Moderate	High	ITS provides higher discriminatory power
Overall marker performance	Stable and conservative	Sensitive and discriminatory	Complementary rather than competing markers

## Data Availability

The accession numbers corresponding to the identified *Rhynchophorus ferrugineus* COI and ITS haplotypes are listed in Table 1, Table 2, Table 3, Table 4, Table 5, Table 6 and Table 7 of the manuscript. All sequence data are freely accessible through the GenBank database.

## Supplementary Materials

The following supporting information can be downloaded at: https://www.mdpi.com/article/10.3390/life16071200/s1. Table S1: Pairewise distance COI; Table S2: Pairewise distance ITS; Table S3. Comparative analysis of similarities and differences between Rhynchophorus ferrugineus haplotypes from Qassim (Saudi Arabia) and global populations; Table S4. Comparative assessment of genetic similarities and differences between Rhynchophorus ferrugineus haplotypes from Qassim (Saudi Arabia) and global reference haplotypes; Table S5. Key genetic distances between selected Rhynchophorus ferrugineus haplotypes from Qassim (Saudi Arabia) and international reference haplotypes; Table S6. Minor genetic distance (<2–4%) between selected Rhynchophorus ferrugineus haplotypes from Qassim (Saudi Arabia) and R. bilineatus (RED1196, KF311740, Papua New Guinea) and R. vulneratus (RED063, KF311716, Indonesia) isolates; Table S7. Major genetic distance (4–>10%) between selected Rhynchophorus ferrugineus haplotypes from Qassim (Saudi Arabia) and R. bilineatus (RED1196, KF311740, Papua New Guinea) and R. vulneratus (RED063, KF311716, Indonesia) isolates.
