# Supplementary material for: Mitochondrial and Nuclear Markers Reveal Contrasting Patterns of Genetic Diversity in the Red Palm Weevil (Rhynchophorus ferrugineus) from Qassim Province, Saudi Arabia
Source: Life (Basel). 2026 Jul 20;16(7):1200. doi: 10.3390/life16071200 (PMC13412979; doi:10.3390/life16071200)
Supplement: Supplementary file 1 [file life-16-01200-s001.zip › life-4385976-supplementary.pdf]

Table S3. Comparative analysis of similarities and differences between *Rhynchophorus ferrugineus* haplotypes from Qassim (Saudi Arabia) and global populations.

Specimen (Haplotype Codes GenBank Accession Numbers)	Q1_ (PP376129)	Q4_ (PP376132)	Q5_ (PP376133)	Q6_ (PP376134)	Q7_ (PP376135)	Q8_ (PP376136)	Q12_ (PP376140)	Q28_ (PP376156)	Q29_ (PP376157)
*R._ferrugineus_isolate_ M315_(HM043673)*_Egypt	0.0000	0.0000	0.0000	0.0000	0.0000	0.0000	0.0000	0.0000	0.0000
R._ferrugineus _isolate_KSA_Al_Ahsa _(KC954632)_ Saudi_Arabia	0.0000	0.0000	0.0000	0.0000	0.0000	0.0000	0.0000	0.0000	0.0000
R._ferrugineus _isolate_ NF36_(HM043696)_ Pakistan	0.0000	0.0000	0.0000	0.0000	0.0000	0.0000	0.0000	0.0000	0.0000
R._ferrugineus _isolate_ NF48_(HM043691)_ UAE	0.0000	0.0000	0.0000	0.0000	0.0000	0.0000	0.0000	0.0000	0.0000
R._ferrugineus _isolate_ Ind77_(MW789117)_ India	0.0372	0.0372	0.0372	0.0372	0.0372	0.0372	0.0372	0.0372	0.0372

Specimen (Haplotype Codes GenBank Accession Numbers)	Q30_ (PP376158)	Q38_ (PP376165)	Q39_ (PP376166)	Q41_ (PP376168)	Q45_ (PP376172)	Q46_ (PP376173)	Q47_ (PP376174)	Q48_ (PP376175)	Q56_ (PP376180)
*R._ferrugineus*_isolate_ M315_(HM043673)_Egypt	0.0000	0.0000	0.0000	0.0000	0.0000	0.0000	0.0000	0.0000	0.0000
R._ferrugineus _isolate_KSA_Al_Ahsa _(KC954632)_ Saudi_Arabia	0.0000	0.0000	0.0000	0.0000	0.0000	0.0000	0.0000	0.0000	0.0000
R._ferrugineus _isolate_ NF36_(HM043696)_ Pakistan	0.0000	0.0000	0.0000	0.0000	0.0000	0.0000	0.0000	0.0000	0.0000
R._ferrugineus _isolate_ NF48_(HM043691)_ UAE	0.0000	0.0000	0.0000	0.0000	0.0000	0.0000	0.0000	0.0000	0.0000
R._ferrugineus _isolate_ Ind77_(MW789117)_ India	0.0372	0.0372	0.0372	0.0372	0.0372	0.0372	0.0372	0.0372	0.0372

Table S4. Comparative assessment of genetic similarities and differences between *Rhynchophorus ferrugineus* haplotypes from Qassim (Saudi Arabia) and global reference haplotypes

Specimen (Haplotype Codes GenBank Accession Numbers)	Q2_ (PP376130)	Q9_ (PP376137)	Q10_ (PP376138)	Q13_ (PP376141)	Q14_ (PP376142)	Q15_ (PP376143)	Q18_ (PP376146)	Q22_ (PP376150)	Q27_ (PP376155)
***R._ferrugineus*_isolate_ M315_(HM043673)_Egypt**	0.0043	0.0021	0.0021	0.0021	0.0043	0.0021	0.0064	0.0043	0.0021
***R._ferrugineus* _isolate_KSA_Al_Ahsa _(KC954632)_Saudi_Arabia**	0.0043	0.0021	0.0021	0.0021	0.0043	0.0021	0.0064	0.0043	0.0021
***R._ferrugineus* _isolate_ NF36_(HM043696)_Pakistan**	0.0043	0.0021	0.0021	0.0021	0.0043	0.0021	0.0064	0.0043	0.0021
***R._ferrugineus* _isolate_ NF48_(HM043691)_UAE**	0.0043	0.0021	0.0021	0.0021	0.0043	0.0021	0.0064	0.0043	0.0021
***R._ferrugineus* _isolate_ Ind77_(MW789117)_India**	0.0417	0.0394	0.0394	0.0394	0.0417	0.0394	0.0439	0.0417	0.0394
**Specimen (Haplotype Codes GenBank Accession Numbers)**	**Q31_ (PP376159)**	**Q35_ (PP376163)**	**Q36_ (PP376164)**	**Q40_ (PP376167)**	**Q42_ (PP376169)**	**Q43_ (PP376170)**	**Q44_ (PP376171)**	**Q49_ (PP376176)**	**Q55_ (PP376179)**
***R._ferrugineus*_isolate_ M315_(HM043673)_Egypt**	0.0021	0.0086	0.0021	0.0021	0.0064	0.0021	0.0021	0.0021	0.0079
***R._ferrugineus* _isolate_KSA_Al_Ahsa _(KC954632)_Saudi_Arabia**	0.0021	0.0086	0.0021	0.0021	0.0064	0.0021	0.0021	0.0021	0.0079
***R._ferrugineus* _isolate_ NF36_(HM043696)_Pakistan**	0.0021	0.0086	0.0021	0.0021	0.0064	0.0021	0.0021	0.0021	0.0079
***R._ferrugineus* _isolate_ NF48_(HM043691)_UAE**	0.0021	0.0086	0.0021	0.0021	0.0064	0.0021	0.0021	0.0021	0.0079
***R._ferrugineus* _isolate_ Ind77_(MW789117)_India**	0.0394	0.0462	0.0394	0.0394	0.0439	0.0394	0.0394	0.0394	0.0405

Table S5. Key genetic distances between selected *Rhynchophorus ferrugineus* haplotypes from Qassim (Saudi Arabia) and international reference haplotypes.

Specimen (Haplotype Codes GenBank Accession Numbers)	Q16_ (PP376159)	Q23_ (PP376163)	Q26_ (PP376164)	Q32_ (PP376167)	Q51_ (PP376169)	Q57 (PP376170)
***R._ferrugineus*_isolate_ M315_(HM043673)_Egypt**	0.0416	0.0646	0.0699	0.0599	0.1175	0.1964
***R._ferrugineus* _isolate_KSA_Al_Ahsa _(KC954632)_Saudi_Arabia**	0.0416	0.0646	0.0699	0.0599	0.1175	0.1964
***R._ferrugineus* _isolate_ NF36_(HM043696)_Pakistan**	0.0416	0.0646	0.0699	0.0599	0.1175	0.1964
***R._ferrugineus* _isolate_ NF48_(HM043691)_UAE**	0.0416	0.0646	0.0699	0.0599	0.1175	0.1964
***R._ferrugineus* _isolate_ Ind77_(MW789117)_India**	0.0811	0.1053	0.1077	0.1003	0.1613	0.2423

Table S6. Minor genetic distance (<2–4%) between selected *Rhynchophorus ferrugineus* haplotypes from Qassim (Saudi Arabia) and *R. bilineatus* (RED1196, KF311740, Papua New Guinea) and *R. vulneratus* (RED063, KF311716, Indonesia) isolates.

Specimen (Haplotype Codes GenBank Accession Numbers)		Q1_ (PP376129)	Q2_ (PP376130)	Q4_ (PP376132)	Q5_ (PP376133)	Q6_ (PP376134)	Q7_ (PP376135)	Q8_ (PP376136)	Q9_ (PP376137)	Q10_ (PP376138)
*Rhynchophorus_ bilineatus_* isolate_ RED1196_ (KF311740) _Papua_New_Guinea and *Rhynchophorus_ vulneratus_isolate_ RED063_(KF311716)_ Indonesia*	***R. b***	0.0275	0.0322	0.0275	0.0275	0.0275	0.0275	0.0275	0.0299	0.0299
	R. v.	0.0239	0.0283	0.0239	0.0239	0.0239	0.0239	0.0239	0.0261	0.0261
		**Q12_ (PP376140)**	**Q13_ (PP376141)**	**Q14_ (PP376142)**	**Q15_ (PP376143)**	**Q18_ (PP376146)**	**Q22_ (PP376150)**	**Q27_ (PP376155)**	**Q28_ (PP376156)**	**Q29_ (PP376157)**
	R. b	0.0275	0.0299	0.0322	0.0299	0.0346	0.0322	0.0299	0.0275	0.0275
	R. v.	0.0239	0.0261	0.0283	0.0261	0.0305	0.0283	0.0261	0.0239	0.0239
		**Q30_ (PP376158)**	**Q31_ (PP376159)**	**Q35_ (PP376163)**	**Q36_ (PP376164)**	**Q38_ (PP376165)**	**Q39_ (PP376166)**	**Q40_ (PP376167)**	**Q41_ (PP376168)**	**Q42_ (PP376169)**
	R. b	0.0275	0.0299	0.0370	0.0299	0.0275	0.0275	0.0299	0.0275	0.0346
	R. v.	0.0239	0.0261	0.0328	0.0261	0.0239	0.0239	0.0261	0.0239	0.0305
		**Q43_ (PP376170)**	**Q44_ (PP376171)**	**Q45_ (PP376172)**	**Q46_ (PP376173)**	**Q47_ (PP376174)**	**Q48_ (PP376175)**	**Q49_ (PP376176)**	**Q55_ (PP376179)**	**Q56_ (PP376180)**
	R. b	0.0299	0.0299	0.0275	0.0275	0.0275	0.0275	0.0299	0.0312	0.0275
	R. v.	0.0261	0.0261	0.0239	0.0239	0.0239	0.0239	0.0261	0.0282	0.0239

Table S7. Major genetic distance (4–>10%) between selected *Rhynchophorus ferrugineus* haplotypes from Qassim (Saudi Arabia) and *R. bilineatus* (RED1196, KF311740, Papua New Guinea) and *R. vulneratus* (RED063, KF311716, Indonesia) isolates.

*Rhynchophorus_ bilineatus_* isolate_RED1196_ (KF311740) _Papua_New_Guinea and *Rhynchophorus_ vulneratus_isolate_ RED063_(KF311716)_Indonesia*	**Genetic diversity (4–10%) among *R. ferrugineus* haplotypes**												
	**Specimen (Haplotype Codes GenBank Accession Numbers)**	**Q3 (PP376131)**	**Q11_ (PP376139)**	**Q16_ (PP376144)**	**Q19_ (PP376147)**	**Q20_ (PP376148)**	**Q21_ (PP376149)**	**Q23_ (PP376151)**	**Q25 (PP376153)**	**Q26_ (PP376154)**	**Q32_ (PP376160)**	**Q33_ (PP376161)**	**Q34 (PP376162)**	**_Ind77 _(MW789117)-_India**
	R. b	0.0489	0.0465	0.0709	0.0513	0.0441	0.0440	0.0910	0.0417	0.0961	0.0909	0.056	0.0489	0.0635
	R. v.	0.0440	0.0418	0.0671	0.0463	0.0418	0.0438	0.0908	0.0373	0.0932	0.0860	0.0509	0.0440	0.0624
	**High genetic diversity (>10%) among *R. ferrugineus* haplotypes**												
	**Specimen (Haplotype Codes GenBank Accession Numbers)**	**Q17_ (PP376145)**	**Q24_ (PP376152)**	**Q51_ (PP376177)**	**Q54_ (PP376178)**	**Q57 (PP376181)**							
	R. b	0.1328	0.1169	0.1464	0.2552	0.2184							
	R. v.	0.1279	0.1154	0.1381	0.2487	0.2140							
